# William Matthew Warren

**Published:** 1904-01

**Authors:** 


William Matthew Warren, of Detroit, business manager for the house of Parke, Davis & Company, died November 11, 1903, aged 39 years. He entered the service of the firm at the age of 17 years, served in the various grades until at thirty-two he became general manager. He was a man of rare gifts and attached himself strongly to his employers and subordinates. The directors of the company adopted a touchingly tender memorial and have sent it out in appropriate form.

Mr. Warren was the publisher of the several medical journals in which Parke, Davis & Company are interested,—Medicine, The Medical Age and the Therapeutic Gazette.

